# How can the practice nurse be more involved in the care of the chronically ill? The perspectives of GPs, patients and practice nurses

**DOI:** 10.1186/1471-2296-7-14

**Published:** 2006-03-03

**Authors:** Thomas Rosemann, Katharina Joest, Thorsten Körner, Rainer Schaefert, Marc Heiderhoff, Joachim Szecsenyi

**Affiliations:** 1 Department of General Practice and Health Services Research, University of Heidelberg, Voβstr. 2, 69115 Heidelberg, Germany; 2 Center of Psychosocial Medicine, Clinic of Psychiatry, University of Heidelberg, Voβstr. 4, 69115 Heidelberg, Germany; 3 Center for Psychosocial Medicine, Clinic of General Adult Psychiatry, Dept. of Psychosomatic Medicine, University of Heidelberg, Thibautstr. 2, 69115 Heidelberg, Germany

## Abstract

**Background:**

A well established "midlevel" of patient care, such as nurse practitioners and/or physician assistants, exits in many countries like the US, Canada, and Australia.

In Germany, however there is only one kind of profession assisting the physician in practices, the practice nurse. Little is known about the present involvement of practice nurses in patients' care in Germany and about the attitudes of GPs, assistants and patients concerning an increased involvement. The aim of our study was to get qualitative information on the extent to which practice nurses are currently involved in the treatment of patients and about possibilities of increased involvement as well as on barriers of increased involvement.

**Methods:**

We performed qualitative, semi-structured interviews with 20 GPs, 20 practice nurses and 20 patients in the Heidelberg area. The interviews were digitally recorded, transcribed and content-analysed with ATLAS.ti.

**Results:**

Practice nurses are only marginally involved in the treatment of patients. GPs as well as patients were very sceptical about increased involvement in care. Patients were sceptical about nurses' professional background and feared a worsening of the patient doctor relationship. GPs also complained about the nurses' deficient education concerning medical knowledge. They feared a lack of time as well as a missing reimbursement for the efforts of an increased involvement. Practice nurses were mostly willing to be more involved, regarding it as an appreciation of their role. Important barriers were lack of time, overload with administrative work, and a lack of professional knowledge.

**Conclusion:**

Practice nurses were only little involved in patient care. GPs were more sceptical than patients regarding an increased involvement. One possible area, accepted by all interviewed groups, was patient education as for instance dietary counselling. New treatment approaches as the chronic care model will require a team approach which currently only marginally exists in the German health care system. Better medical education of practice nurses is indispensable, but GPs also have to accept that they cannot fulfil the requirement of future care alone.

## Background

In Germany medical care in the primary care setting is exclusively provided by a physician, the GP. In a normal practice, several practice nurses support the physician mainly in administrative things like arranging appointments for patients, answering telephone calls, preparing and providing the patient files and so on.

However, technological and/or societal change especially concerning the health care system is stimulating consideration of expanding nursing roles in Germany. For example, due to a big problem in prospective candidates, especially in primary care, the number of GPs in Germany will drop tremendously in upcoming years. In some rural areas, especially in the eastern part of Germany, it is already very difficult to find young GPs who are willing to work there. Additionally, the workload is increasing due to administrative work. The newly introduced disease management programs (DMPs) have again boosted this trend. This aggravation of labour conditions has started a fatal circle: more and more German physicians are leaving the country, mostly to work in the U.K. or Scandinavian countries where they find better working conditions [1,2]. Thus, these developments force physicians and policymakers to consider new models of nurses' involvement.

In other countries, different health care professionals are involved in patient's care. In the U.S. for example from the 1960s on, physicians assistants were established, providing care together with nurses and nurse practitioners [3,4]. There is very little equivalence in education and roles across borders. Different roles of nurse practitioners (NP) and physician assistants (PA) exists in different countries. In the United States, both of these roles generally require graduate education involving intensive study in diagnostic methods and therapeutics. However, several studies showed that involvement of medical assistants improves patient care or quality of life, even if the involvement contained simple procedures as for instance frequent contacts by telephone calls [5,6]. Therefore, the number of assistants increased constantly in the primary care setting in Canada as well as in the U.S.

Different to the U.S. and Canada, little is known about the involvement of doctors' assistants in the care of patients in Germany. GPs are complaining about increasing workload so that increased involvement of doctors assistants could reduce the workload and help them concentrate on patients. Especially, chronic diseases with frequent consultations and less change in therapy could be possible domains where an increased involvement of practice nurses decreases GPs' workload and increases patient satisfaction [5]. Chronic diseases often require knowledge about coping strategies as well as information on strategies to prevent further deterioration. This is reflected in the Chronic Care Model (CCM), a conceptual framework for delivering care for chronically ill, which has received widespread acceptance. However, the implementation of the CCM requires a team approach, i.e. heightened involvement of practice nurses in patient care.

It seems that the time is right to consider a new breed of healthcare professionals, who could take on many of the tasks currently undertaken by doctors and therefore enable the physicians to concentrate on their original duty: providing medical care.

The aim of our study therefore, was to assess the present involvement of practice nurses in patient care, to estimate possible areas of heightened involvement and to reveal existing barriers by exploring the perspectives of all groups involved in the treatment process: patients, GPs and practice nurses.

## Methods

We chose a qualitative approach because little is known about involvement of practice nurses in the care of chronically ill patients. So far, there has been no German study on this topic.

### Sample

The selected GPs, assistants and patients represented a stratified sample regarding gender, city and rural population living in Heidelberg and surrounding areas [7]. The GPs were to have a minimum of 5 years experience; the practice nurses were required to have a minimum of 10 years professional experience. The patients were selected at random from the GPs' computer files. They had osteoarthritis as primary chronic disease and all of them had at least one additional chronic disease such as diabetes, heart insufficiency or hypertension. During their practice visit the GP asked whether they were willing to participate in an interview. All patients but one agreed to take part in the study. Written consent of all participants was obtained. The study was approved by the ethics committee of the University of Heidelberg; approval number 019/2004.

### Interviews

The interviews were conducted in summer 2004. The GPs and assistants were interviewed in their respective practices; the patients were interviewed at home by a trained interviewer. After a detailed study of the literature regarding patients' perspectives on chronic diseases, we compiled open-question interview guidelines. In order to have the possibility to compare the views of GPs, patients and practice nurses, we matched the interviews for the three groups on important issues but also asked specific questions concerning only the investigated group. The questions focused on the following aspects:

• Actual areas of practice nurses' involvement

• Main obstacles regarding more involvement

• Possibilities to overcome the obstacles

Following the regular process of care, these aspects were assessed in the following areas:

• diagnostic procedures, examinations and treatment

• advice giving / counselling

• referrals

During the interview, the interviewer ensured that every aspect was explained sufficiently and in detail, so that no questions or misunderstandings remained.

### Data analysis

The interviews were recorded digitally, transcribed literally and analysed by four different researchers with ATLAS.ti -Software [8]. In advance, a categorising system had been established based on the interview guidelines. In order to achieve maximum objectivity, all interviews were read by all researchers and categorised independently. The categorising system was consequently modified after agreement had been obtained among all four researchers. Numerous free categories were developed from the text, discussed and adjusted so that they were as similar as possible in all three interviewed groups, since the objective was to compare the different perspectives of the groups.

## Results

Although the interview guidelines for all groups contained the same number of questions, the interviews differed in length depending on the group, with the GPs' interviews being the longest and the assistants' interviews being the shortest (Table 1). The categorical systems with subcategories are displayed for each interviewed group in tables 2, 3, 4. The numbers in brackets display how many participants responded to the respective category.

**Table 1:** Baseline characteristics of the study sample

	Practice nurses	GPs	Patients
N	20	20	20
Mean age (range)	41.3 (29–56)	43.5 (33–57)	56 (40–78)
Years of working experience in general practice (range, mean)	13–35 (21.7)	8–19 (11.3)	
Longest duration of chronic disease (Mean/ (SD))			17 (9.3)
Number of chronic diseases (Mean/(SD))			2.9 (1.1)

**Table 2:** Categorical system with first subcategories (General Practitioners)

Main categories	First subcategory
Present situation (20*):	• Involvement in medical proceedings (6) • No involvement in medical proceedings (14)
*Team approach in general (20):*	• Imaginable (18) • Not imaginable (2)
*Barriers / Problems (20):*	• Lacking (medical) knowledge/skills (19) • Workload (16) • Perceived lacking patients' acceptance (14) • Fear of worsening physician-patient-relationship (12) • Lacking reimbursement (5) • Doubt about efficacy of increased involvement (5) • Lacking motivation by practice nurses (2)
*Possible tasks for practice nurses in the context of a team approach (20):*	• Hand out patient information leaflets (12) • Life style counselling/advice giving (11) • Arranging/Assisting referrals (5) • Others (4)
*Possibilities to overcome the obstacles (12):*	• Better education for practice nurses (11) • Reimbursement (5) • Training offers for practice nurses (2)

* numbers in parentheses are the frequency of subjects, who said something to the respective category

**Table 3:** Categorical system with first subcategories (Patients)

*Main categories*	First subcategory
Team approach in general (20*):	• Imaginable (19) • Not imaginable (1)
*Barriers / Problems (20):*	• Lacking (medical) knowledge (11) • Fear of worsening physician-patient- relationship (2) • Others (4)
*Possible tasks for practice nurses in the context of a team approach (13):*	• Organising education groups (10) • Giving information about referrals (specialists) (4) • Asking patient about his mood (2) • Talking to the patient sympathetically (1)

* numbers in parentheses are the frequency of subjects, who said something to the respective category

**Table 4:** Categorical system with first subcategories (Practice Nurses)

*Main categories*	First subcategory
Present situation (20*):	• Involvement in medical proceedings (3) • No involvement in medical proceedings (17)
*Team approach in general (20):*	• Imaginable (20) / Wish of being more involved (18) • Not imaginable (0) / No wish of being more involved (2)
*Barriers / Problems (20):*	• Lacking (medical) knowledge/skills (20) • Workload (17) • Perceived lacking patients' acceptance (1) • Others (4)
*Possible tasks for practice nurses in the context of a team approach (15):*	• Giving information on local offers, self help groups, etc. (13) • Calling the patient in regular intervals (i.e. case management) (7) • Motivating the patient to use self-help groups and social contacts (3) • Organising (self-help) groups (2) • Exchanging information about patient with GP (2)
*Possibilities to overcome the obstacles (19):*	• Improved medical education (14) • Changes in practice organisation (11) • More support by GP (3) • Training offers for practice nurses (2)

* numbers in parentheses are the frequency of subjects, who said something to the respective category

### Involvement in diagnostic procedures, examinations and treatment

GPs considered it adequate to delegate simple routine examinations as for instance the measurement of blood pressure or the measurement of height and weight to the practice nurses. They assumed that patients would accept practice nurses to perform only these examinations. Taking a blood sample was appraised quite differently: Some GPs regarded it as alleviation, some preferred to take the blood themselves. The main reason for these differences was not assumed lack of knowledge or skills but rather the GPs' preferences with respect to the proceedings in the practice.

"I prefer to take the blood myself; I can already start talking to the patient.....Sometimes I get the most important information during this procedure" GP 7

All GPs said that it is their duty to perform the examination, to inform the patient and explain diagnosis, prognosis and therapy. Most GPs were convinced that examinations, the following explanations and counselling represents one of the main challenges in primary care and that this can only be done by the physician. A lack of medical knowledge was also mentioned as an important obstacle against a broader involvement of the practice nurse in this area. The third most frequently named obstacle was that GPs are convinced that patients expect to be informed about diagnosis, prognosis and therapeutic options only by the physician.

„To inform the patients that's only my job. That's also what the patient expects. They would never accept that the practice nurse tells them what's going on with them. " GP 17

Most patients found the practice nurse to be well skilled to measure the blood pressure, blood sugar and also to take i.v. blood samples. Information about the disease itself, the prognosis and possible treatment options were very important to patients. In accordance to the assumption of the GPs, they expected to receive important information about their disease only from the physician. They assumed that the practice nurses do not have the knowledge to inform them about the causes and prognosis of the disease. Moreover, it was very important to the patients that they can always talk directly to the GP about their concerns without having to explain the requests to the practice nurse first. With respect to treatment many patients seemed to assume that the practice nurse is familiar with possible treatments beyond the evidence based procedures. Many patients assumed that the practice nurses have an overview or knowledge about treatments that had been beneficial for other patients. The practice nurse was regarded as a source for additional treatments which are beyond regular treatment procedures.

„Well, I do sometimes ask the practice nurse, if she knows something which may have helped other patients, some sort of cream or something, which maybe the doctor can't prescribe." P3

Practice nurses stated that they are not asked about the diagnosis or prognosis of diseases by the patient. They complained that their schooling focused more on administrative things than on medical knowledge. Due to this, they did not feel competent to say anything about the disease, its cause, possible influences on the progression, the treatment and prognosis. The knowledge they have on these aspects is mostly acquired by working experience and not by schooling. Patients rarely questioned the practice nurse regarding medication. Formal matters such as equivalence of medications with different names etc. were matter of concern. The practice nurses did not consider themselves competent enough to talk about medication and often refer to the GPs' instructions. All of them desired receiving more medical information during their education, but nevertheless they clearly stated that providing this information to the patient is up to the GP. Practice nurses' statements were in line with patients' statements: most of them confirmed that they were frequently asked about additional treatment options which are beyond the classical treatment as for instance possibilities to support a life-style change, supplements, etc.

"Patients often ask me: do you know somebody who had positive experiences with this or that.....sometimes I wonder why they don't ask the doctor about that, but it seems that they feel ashamed to ask the doctor about this, especially the older ones." N 10

"Sometimes I ask the patient if he mentioned the problems to the doctor during the consultation, but the patients often reply that the doctor is too busy and that they don't want to bother him with their complaints to much, because there's no real effective relief anyway." N 19

### Involvement in counselling

As shown in the interviews, advice giving or some sort of counselling by the practice nurse was acceptable for the GPs mainly in the fields of „life-style-change", „nutrition" and „motivation for physical activity". Advice concerning medical issues such as pharmacological treatment or other specific treatments is regarded to be solely GPs' responsibility. Moreover, as GPs indicated, the involvement of practice nurses is possible only in the context of group education for patients, whereas individual guidance is objected for financial and time reasons. In the context of DMP's such educational groups for patients have been implemented in many practices. However, some GPs were ambivalent concerning group education for patients. Some doubted their efficacy, others criticised that it is too time-consuming or that some practice nurses are not motivated enough.

„These diabetes education groups are quite all right, but what if even more DMPs will be implemented? Should the practice nurse educate patients all day long then?" GP6

"...you can communicate that to younger practice nurses, but that doesn't work with older assistants, they just don't regard it to be their duty..." GP13

Most of the GPs found it acceptable that practice nurses hand out patient information leaflets or point out contacts such as self-help groups. However, some GPs indicate that this kind of patient care is already beyond their field of duty.

„She can't assess what is good for the individual patient, and I don't think it's good if the task is handed down to the next level..." GP 10

„We can't coordinate patients' sports activities. " GP14

All practice nurses found involvement in counselling, for example in the context of DMP, to be an appreciation of their work and a diversion from administrative tasks which constitute their daily routine. Some practice nurses objected, that they are only insufficiently qualified for advice giving which highlights the importance of high-quality education for practice nurses. Education programs in the context of DMPs seem to attend to this aspect insufficiently.

Patients were mainly positive about educational groups and think that the practice nurse is competent to offer such groups. In addition, most patients would like the practice nurses to hand out printed information, and provide knowledge about self help groups, community based or other local offers. They regarded the practice nurse to be a more adequate source for this kind of information than the GP.

### Involvement in the referral process

Concerning the referral process, the GPs delegate many steps in the process to the practice nurses, e.g. filling in the referral form or making an appointment in case of urgent referral. The practice nurses were responsible for the administrative part of the referral. Some GPs generated lists of specialists, which the practice nurses hand out to the patients.

Practice nurses indicated, that patients often ask them about recommendable physicians regarding criteria such as localisation, friendliness and short waiting times. Even if lists with specialists are handed out to patients, they often asked for personal recommendations.

„Especially older patients appreciate it when we show them on the map how they can get there or which bus to take." N 5

Patients did appreciate getting information about specialists they can consult. Particularly when the GP does not explicitly recommend a specialist, patients contacted practice nurses because they have a lot of information.

„...it's not that easy to walk anymore, and I'm really glad, when they tell me about an orthopaedist I can go to and reach easily." P19

## Discussion

In German practices, there is only one kind of assistance for the GP, the practice nurse. As our findings showed, practice nurses are currently only rarely involved in diagnostics and treatment and are mainly occupied with administrative tasks. The DMPs as performed in Germany can be largely regarded as management by protocol, meaning that they do not require extensive physicians' involvement. Therefore, since the implementation of DMPs, many practice nurses are more involved in giving patients' advice, which most of them do appreciate. Patients as well as most of the GPs are also positive about this involvement. The growing role of disease management programs have led to considerations regarding nurses' deeper involvement in the care of the chronically ill. For most of these patients the diagnostic phase is largely over, meaning that the more technically sophisticated and often more lucrative phase of care has passed – the "threat" to physicians is minimal. Furthermore, the great deal of time-consuming patient teaching involved in the care of chronically ill patients in maintenance care is believed to "come naturally" to nurses as a result of their education. Additionally, patient teaching can be done in a cheaper way by nurses, thus liberating physicians for "more complex" care.

Major barriers for further integration into care according to practice nurses are professional deficits stemming from a lack of medical contents in their education. Therefore, continuing education for practice nurses is of great importance. However, with regard to further training, the study showed that there is still a lot of room for improvement quantitatively as well as qualitatively.

Regarding physicians, our results also indicated that the expansion of roles will only work if physicians do not feel threatened by the shift of territory and responsibility, believe that heightened involvement of nurses leads to advantages such as easier workload and happier patients and are confident concerning nurses' competence.

Moreover, the study showed that the role of the practice nurse in Germany is very different compared to the role nurse practitioners or physicians assistants have for example in the US or Canada, where they are an essential part of care [9]. This is a prerequisite for the implementation of new treatment approaches as for instance the Chronic Care Model (CCM). The CCM is a recently developed conceptual framework for the care of chronically ill patients, which favours planned and proactive care [10]. Due to the complex approach of care in this model it cannot be performed by the physician alone but requires a team approach [11,12]. Moreover, the wish of patients for more information will increase further [13,14] and resources on the GPs' side will decrease [1]. Thus, in the near future, it seems inescapable to spread care, which is, as our findings showed, problematic since until now, practice nurses in Germany are only marginally involved in patient care. Even by delegating simple tasks nurse delivered interventions can improve patients QoL and reduce costs [15,16]. Practice nurses involvement can therefore range from regular telephone contacts, which reduce costs and heighten patients' satisfaction to more specialized fields of care [17,18]. Consistent with our results, other studies show that practice nurses would appreciate an upgrading of their work within a more team oriented approach [19].

Our study has some weaknesses, e.g. the relatively advanced age of the patients as well as their low level of education. Older people tend to be happier with their GPs [14,20], which could be one of the reasons why we did not get so many concrete suggestions for improvement from patients. A further weakness is that we did not mention ideas for improvement or interventions in the interview guide. Although this was discussed beforehand, we abandoned that idea in order to keep answers as open and honest as possible.

Despite these limitations, to our knowledge our study is the first to consider individual perspectives of patients, GPs'and practice nurses regarding involvement of practice nurses simultaneously. The interview guidelines were developed interdisciplinary, i.e. in cooperation with a psychologist in order to ensure appropriateness for patients with chronic illnesses. Furthermore, four researchers assessed and categorised the qualitative data independently according to stringent guidelines, in order to achieve the highest possible objectivity [7,21].

## Conclusion

In conclusion, higher qualification of practice nurses could contribute to a reduction of GPs workload. This requires qualitatively improved education and further training for practice nurses [22], which would lead to an appreciation of the profession in return. Our study showed that the majority of patients would accept the practice nurse as a competent part of the care team and that GPs' scepticalness is often the main problem with regard to involvement of practice nurses. This however, will leave an important resource unused and will enlarge the gap between the German health care system and more team-oriented systems.

## Competing interests

The author(s) declare that they have no competing interests.

## Authors' contributions

TR conceived and performed the study and drafted the manuscript. KJ, TK, MH, RH analysed the data and contributed substantially to the manuscript. JS participated in the study design. All authors read and approved the final manuscript.

## Pre-publication history

The pre-publication history for this paper can be accessed here:


